# Supplementary material for: Synthesis of Alginate Nanogels with Polyvalent 3D Transition Metal Cations: Applications in Urease Immobilization
Source: Polymers (Basel). 2022 Mar 22;14(7):1277. doi: 10.3390/polym14071277 (PMC9002911; doi:10.3390/polym14071277)
Supplement: Supplementary file 1 [file polymers-14-01277-s001.zip › polymers-1400770-supplementary.pdf]

Supplementary Materials

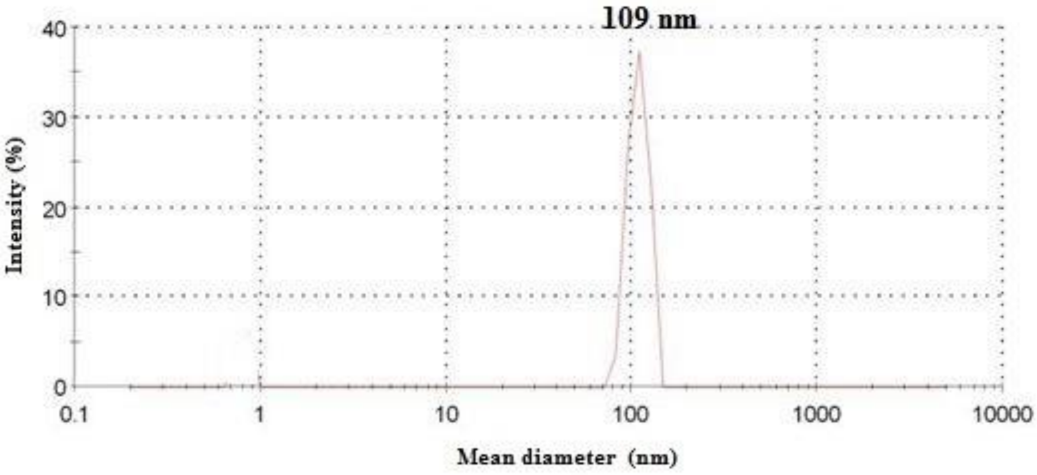

Figure S1. DLS of W/O micro-emulsion of alginate-sol/Hexane/Span 20 representing hydrodynamic diameter of sodium alginate droplet. .

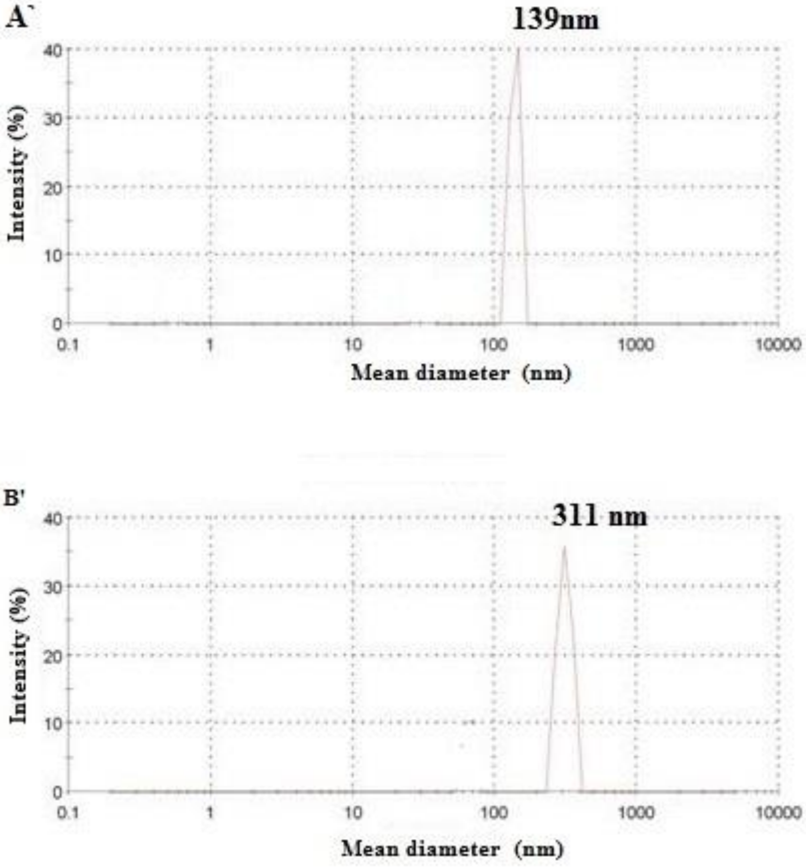

.

C`

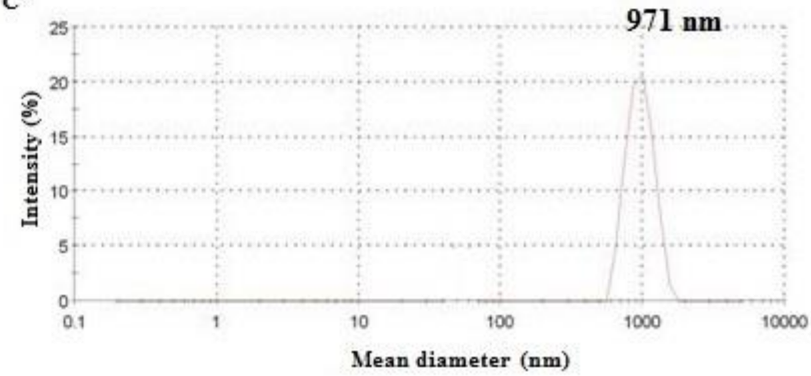

Figure S2. DLS of W/O emulsion of alginate-sol/Hexane/Span 20 representing A`) clear solution; B`) slightly turbid; and C`) milky appearance .

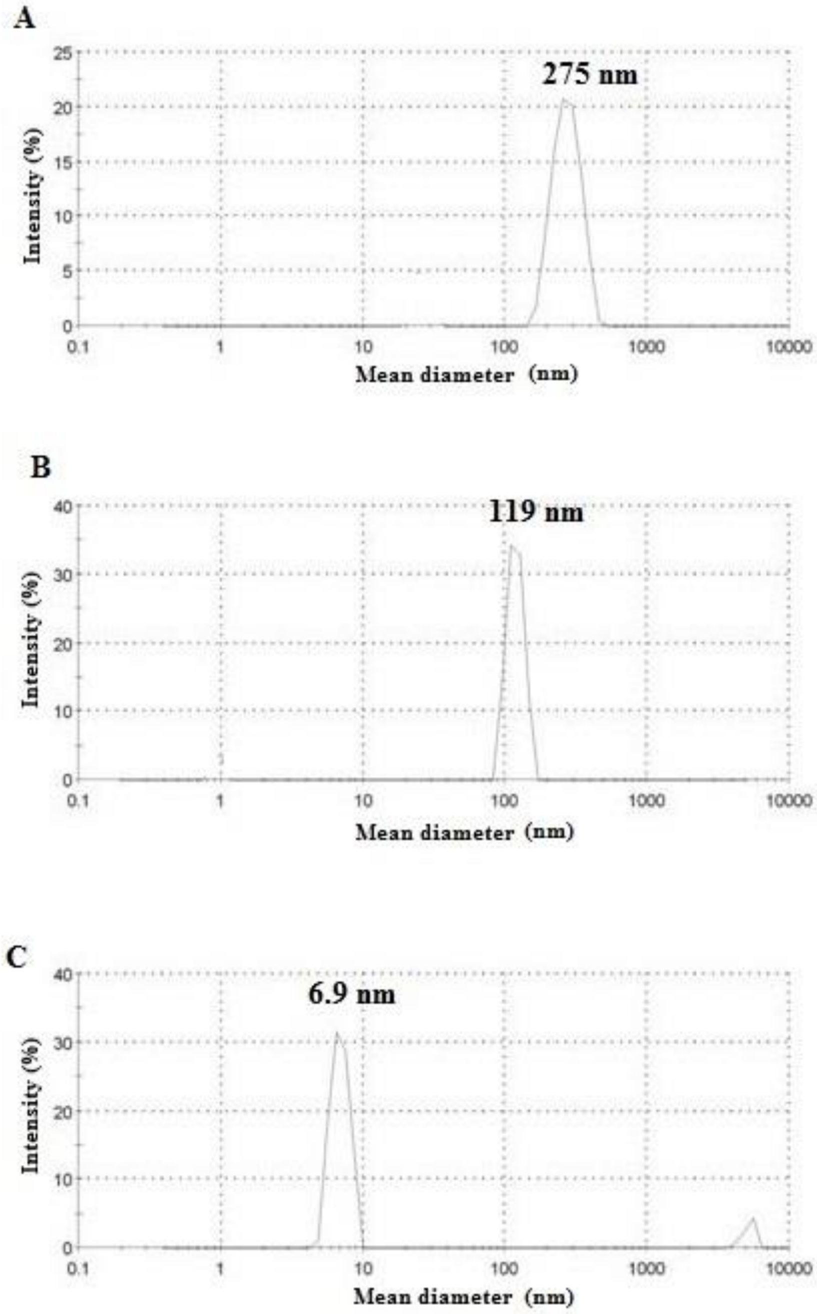

Figure S3. DLS measurement data of: A) Mn-alginate nanogel; B) Fe-alginate nanogel; C) Co-alginate nanogel.

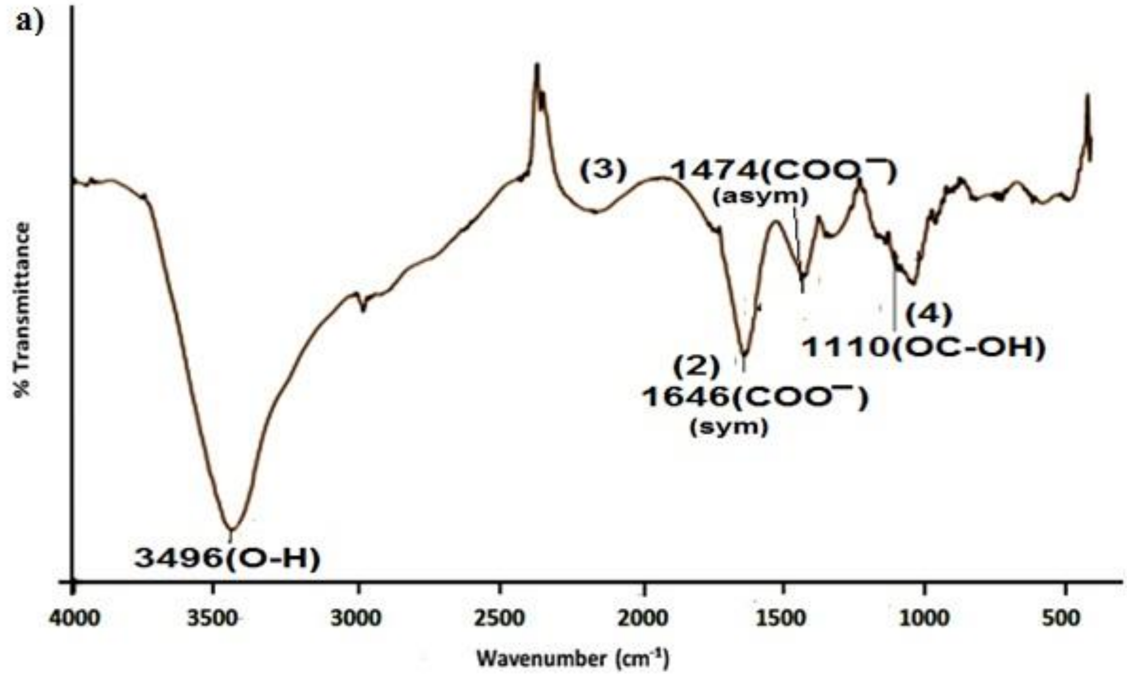

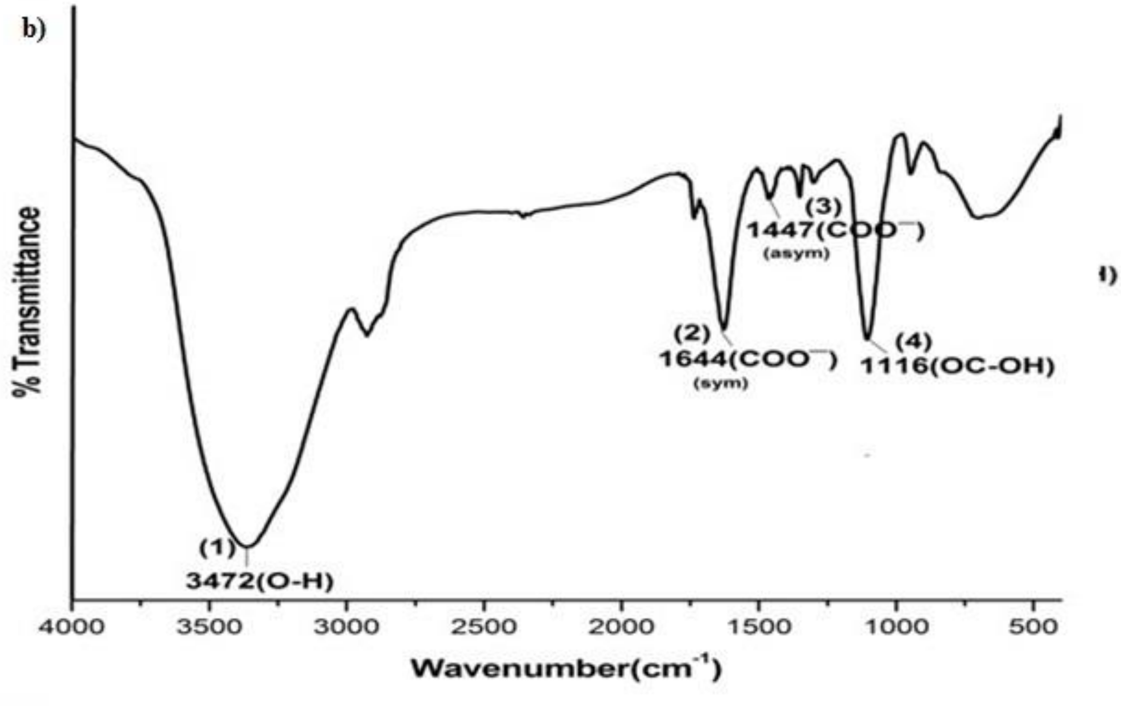

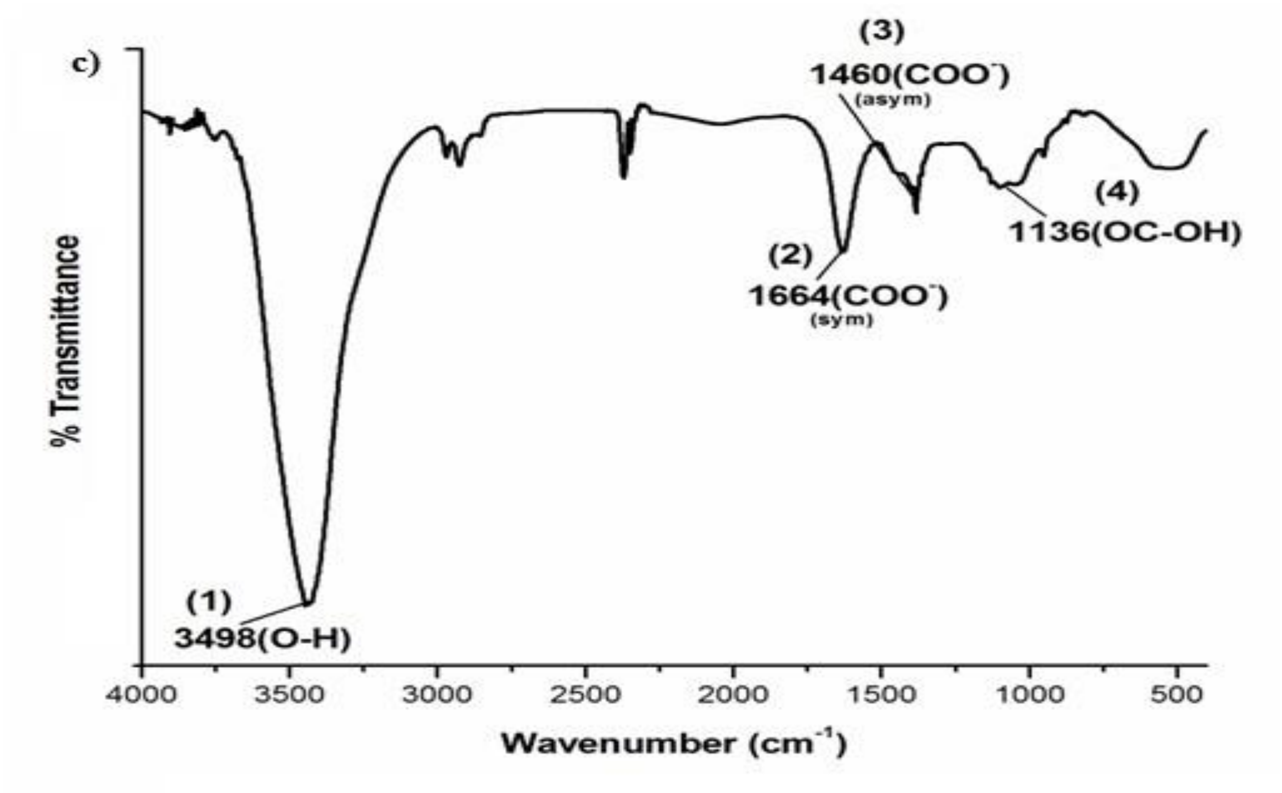

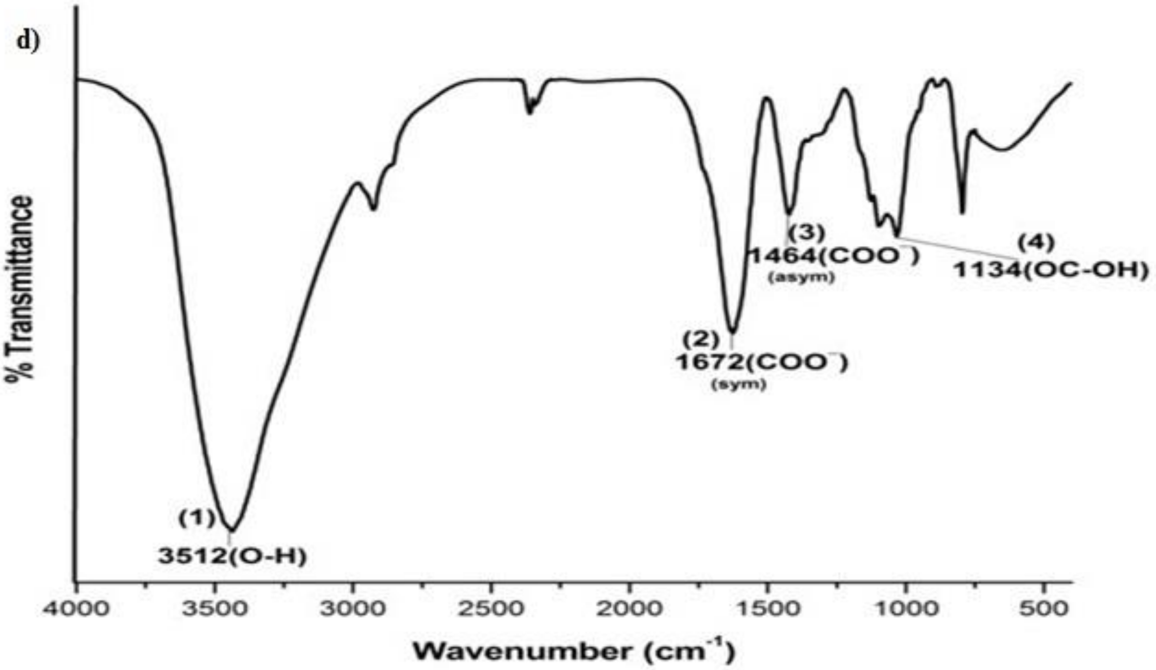

Figure S4. FTIR spectra of (a) alginate salt (sodium alginate) and nanogels cross-linked with: (b)Mn^{+2}, (c) Fe^{+2}, and (d) Co^{+2}.

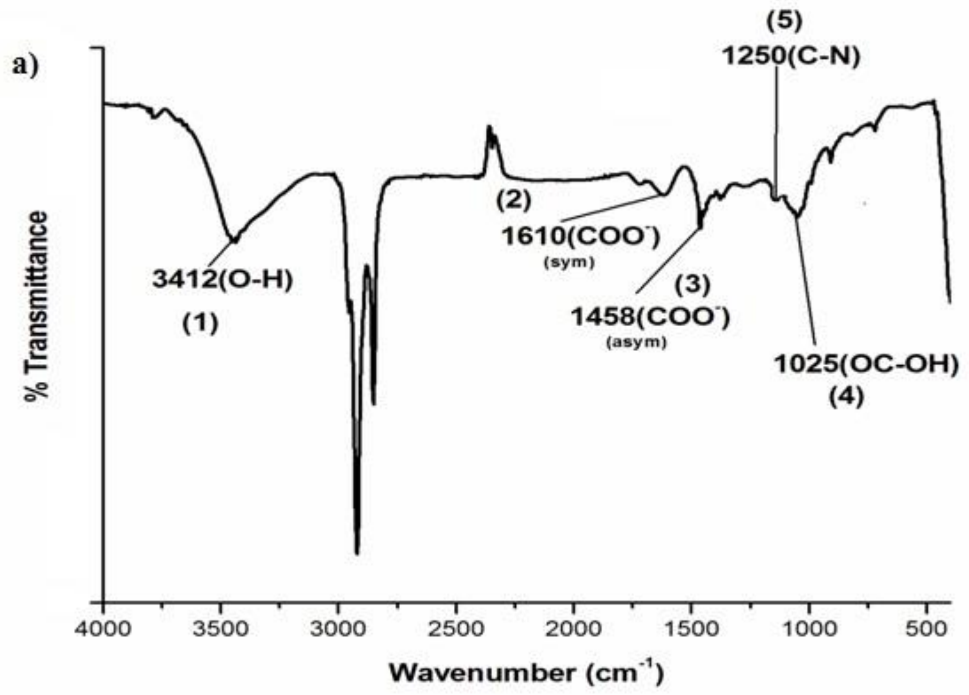
a)
% Transmittance
Wavenumber (cm⁻¹)
4000 3500 3000 2500 2000 1500 1000 500
3412(O-H)
(1)
(2)
1610(COO⁻)
(sym)
(3)
1458(COO⁻)
(asym)
(5)
1250(C-N)
1025(OC-OH)
(4)

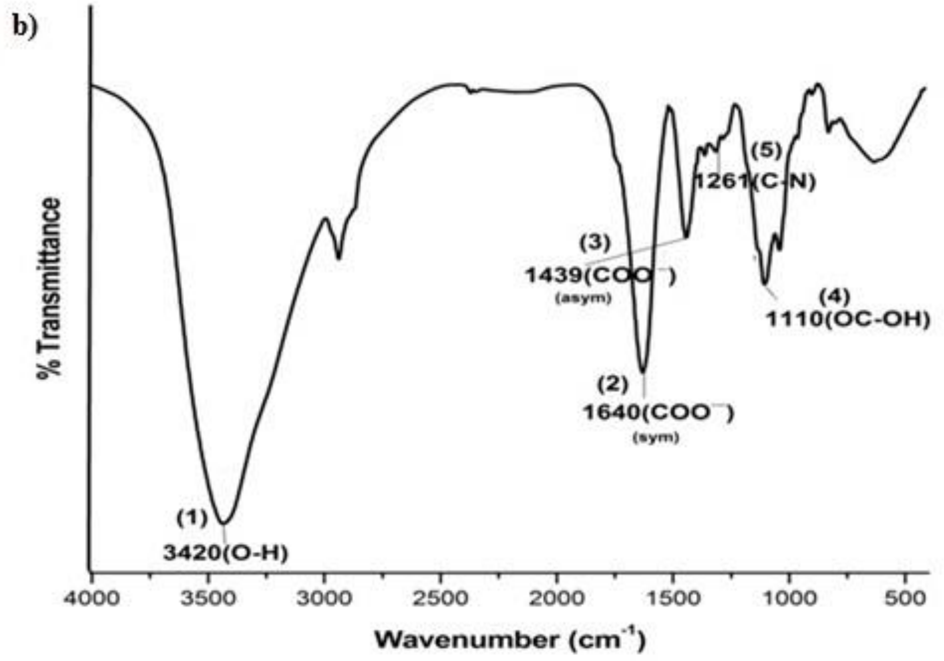
b)
% Transmittance
Wavenumber (cm⁻¹)
4000 3500 3000 2500 2000 1500 1000 500
(1)
3420(O-H)
(2)
1640(COO⁻)
(sym)
(3)
1439(COO⁻)
(asym)
(5)
1261(C-N)
(4)
1110(OC-OH)

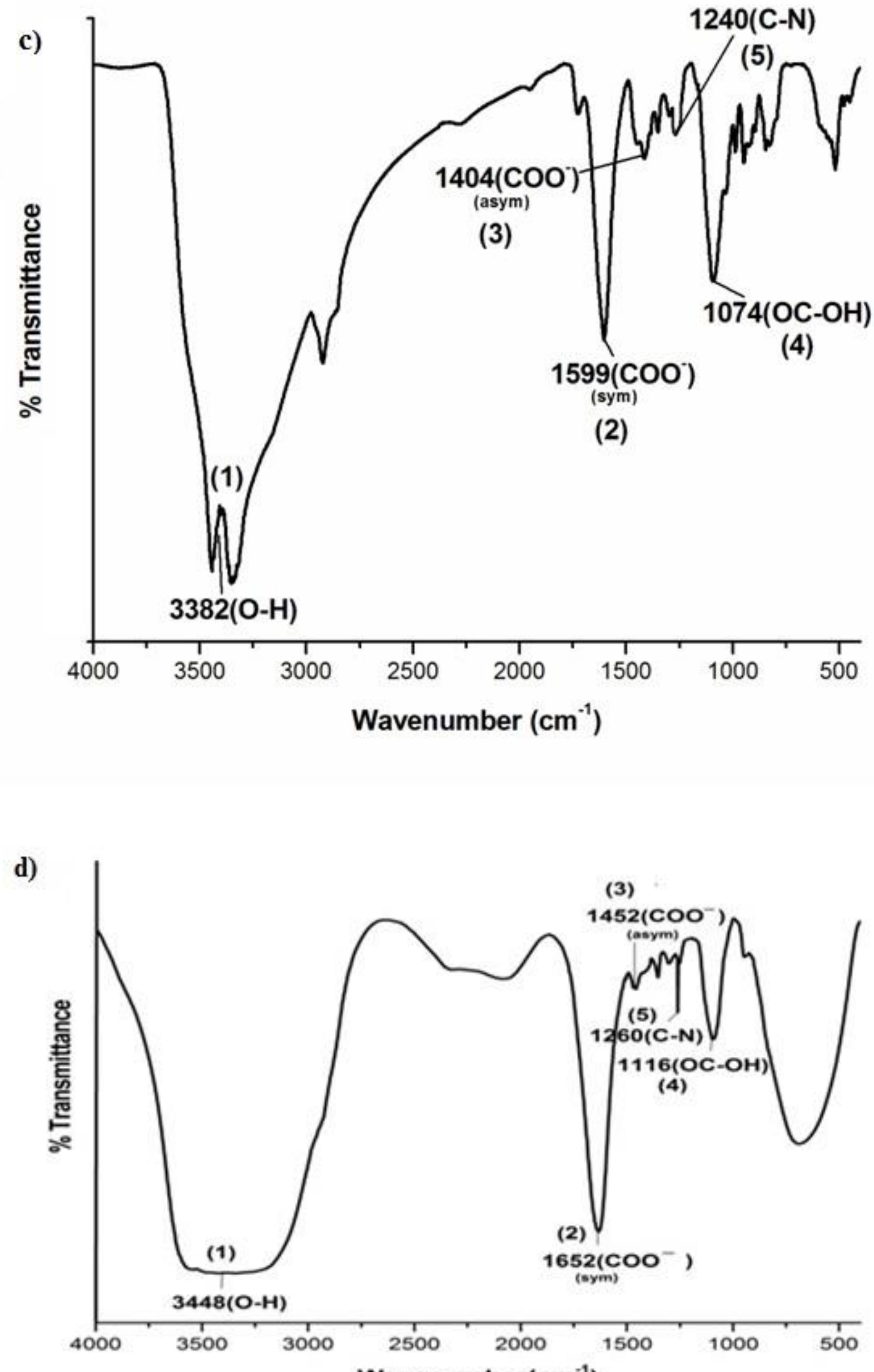

Figure S5. FTIR spectra of (a) urease (lyophilized) and urease encapsulated alginate nanogels formed by cross-linking cations (b) Mn^{+2}, (c)Fe^{+2}, and (d) $Co^{+2.}$

a)

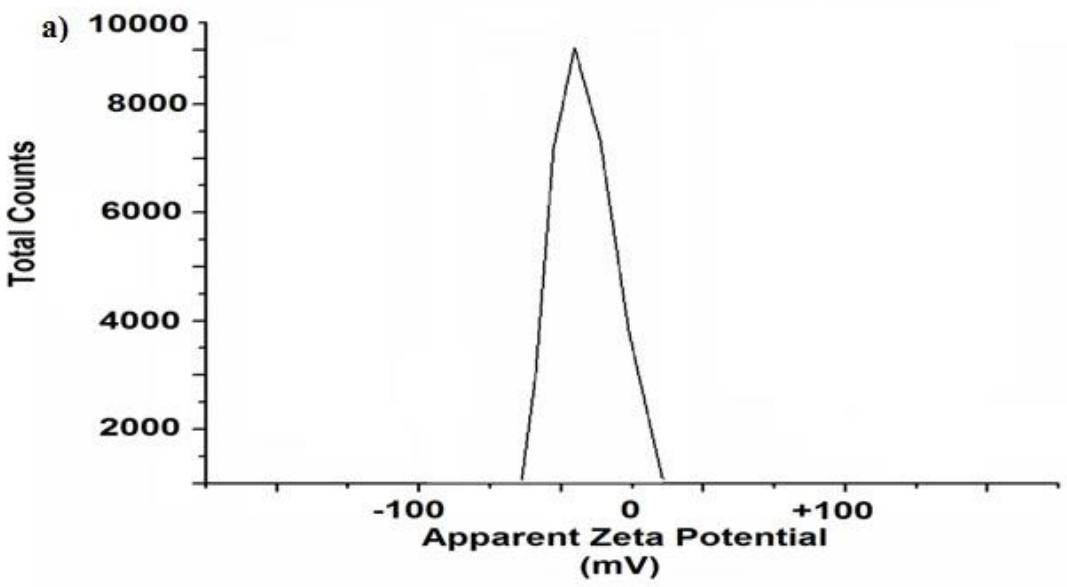
10000
8000
6000
4000
2000
Total Counts
-100
0
+100
Apparent Zeta Potential
(mV)

b)

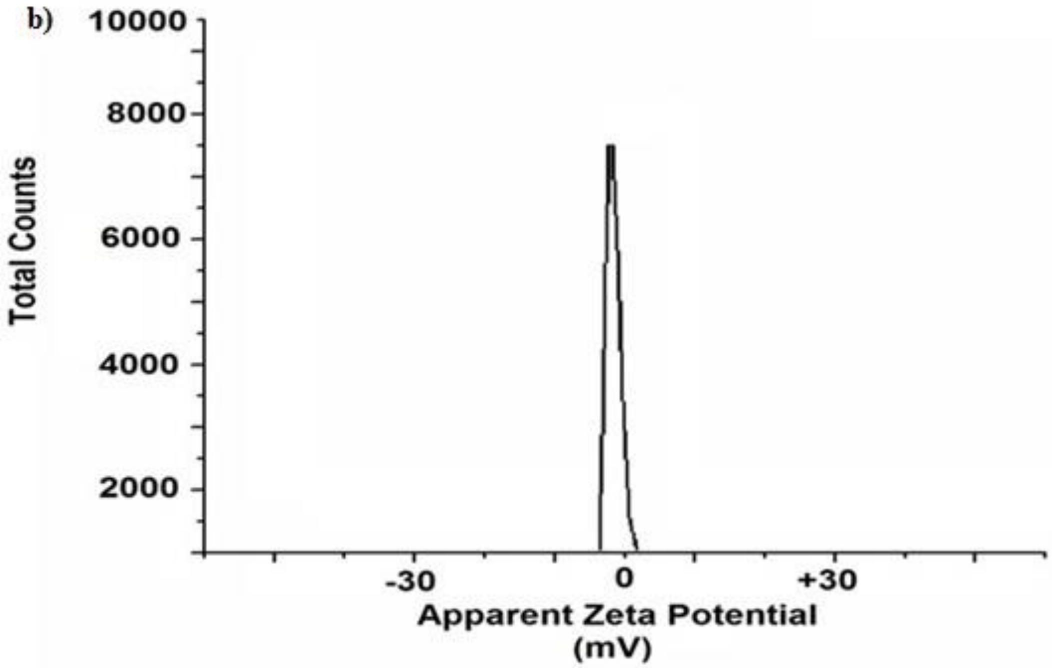
10000
8000
6000
4000
2000
Total Counts
-30
0
+30
Apparent Zeta Potential
(mV)

c)

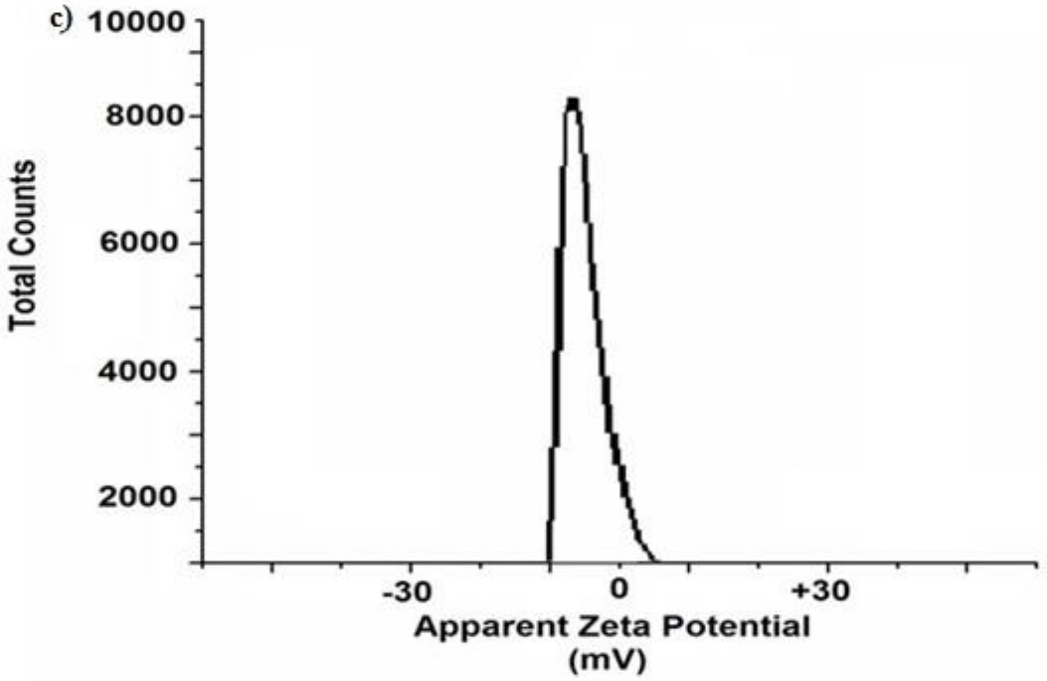

d)

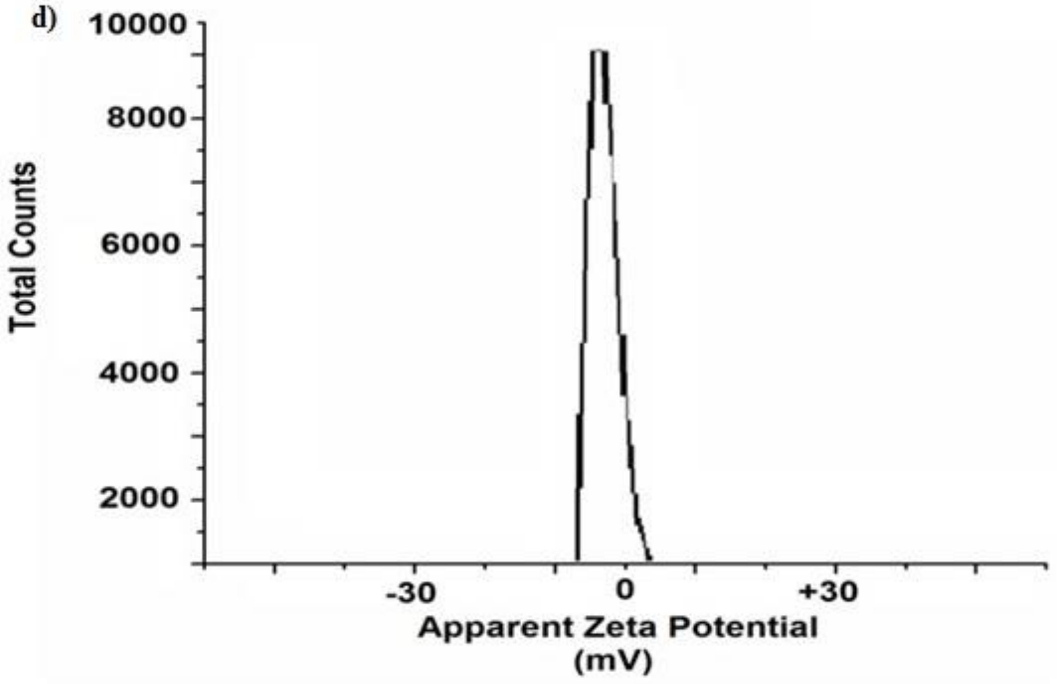

Figure S6. Zeta potentials of (a) sodium alginate (salt) and alginate nanogels crosslinked with: (b) Mn^{2+}, (c) Fe^{2+} , and (d) $Co^{2+.}$

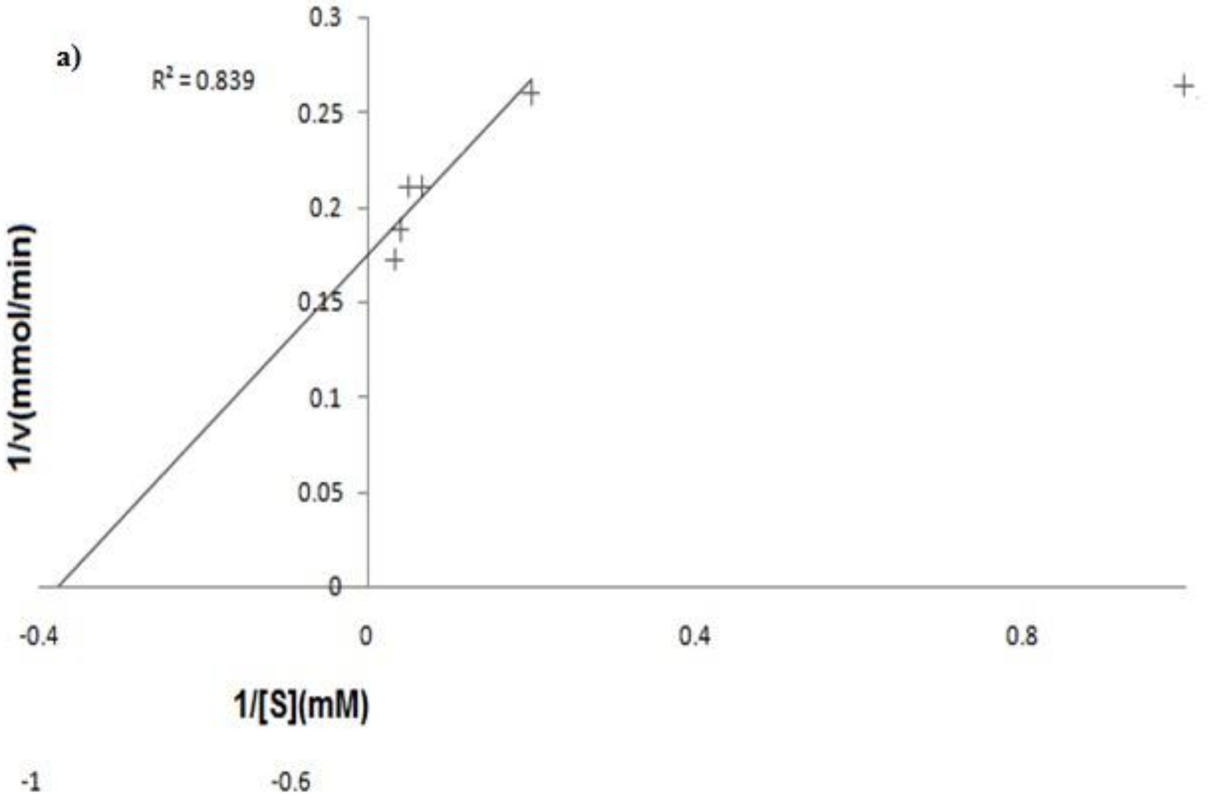
a)
R² = 0.839
1/v(mmol/min)
0.3
0.25
0.2
0.15
0.1
0.05
0
-0.4
0
0.4
0.8
1/[S](mM)
-1
-0.6

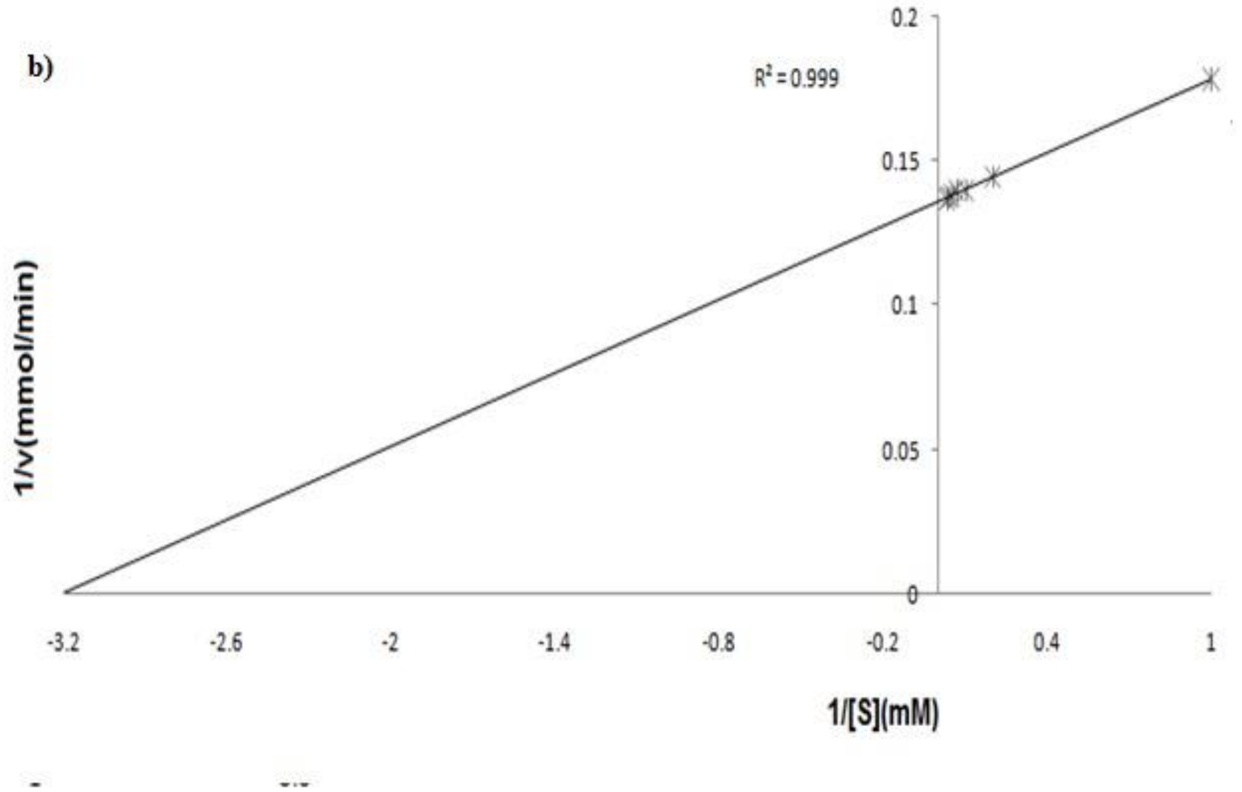
b)
R² = 0.999
1/v(mmol/min)
0.2
0.15
0.1
0.05
0
-3.2
-2.6
-2
-1.4
-0.8
-0.2
0.4
1
1/[S](mM)

c)

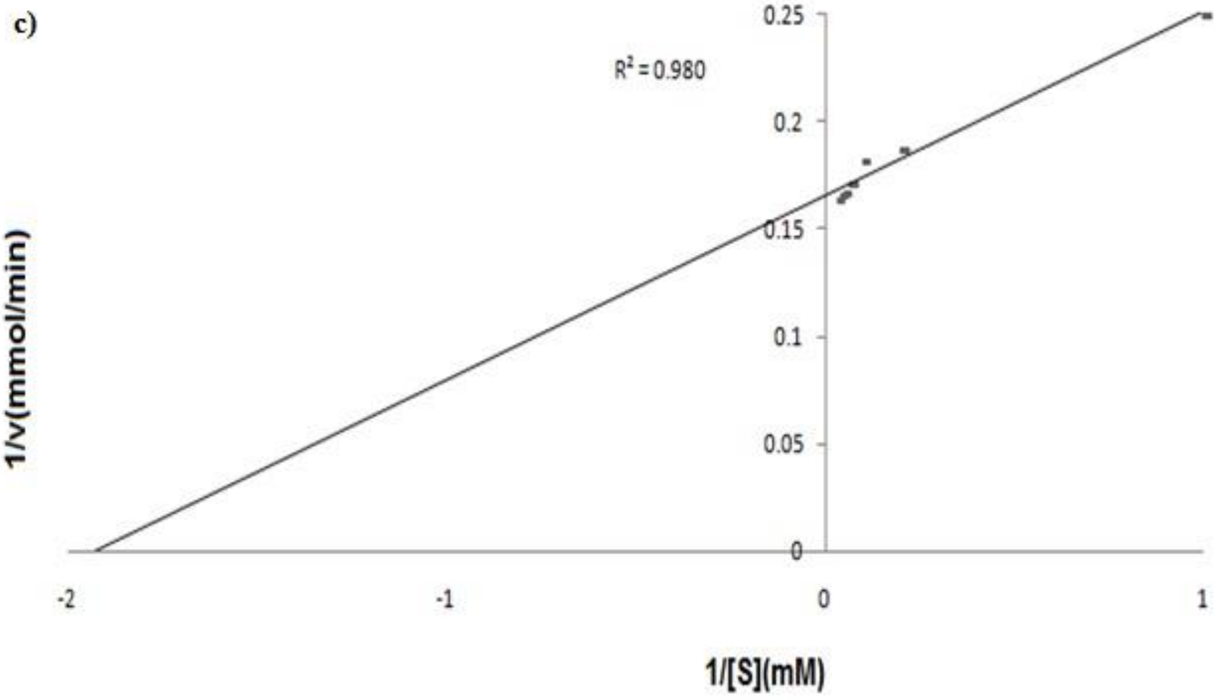

Figure S7. The Lineweaver burk plot obtained at enzyme conc. of 5mg/ml in urease
encapsulated alginate nanogels crosslinked with: (a) Mn^{2+}, (b) Fe^{2+}, and (c) $Co^{2+.}$
